# Microbiological mechanisms of oral mucosal disease: oral-intestinal crosstalk and probiotic therapy

**DOI:** 10.3389/froh.2025.1485028

**Published:** 2025-08-21

**Authors:** Qi Zhou, Panpan Liu, Muni Chen, Hao He, Mengting Xu, Qianrong Xu, Jianfeng Yu, Yutian Deng, Jiayu Yan, Yueqiang Wen

**Affiliations:** ^1^ School of Clinical Medicine, Chengdu University of Traditional Chinese Medicine, Chengdu, China; ^2^ Department of Stomatology, Sichuan Integrated Traditional and Western Medicine Hospital, Chengdu, China; ^3^ Department of Stomatology, Affiliated Hospital of Chengdu University of Traditional Chinese Medicine, Chengdu, China; ^4^ School of Basic Medicine, Chengdu University of Traditional Chinese Medicine, Chengdu, China

**Keywords:** oral microbiota, intestinal microbiota, oral-intestinal axis, oral mucosal disease, probiotics

## Abstract

Balance of the oral-intestinal axis microbiota is essential for maintaining oral mucosal health. The occurrence of oral disease is closely linked to the microbiota, this disorder is closely related to the pathogenesis of oral mucosal diseases, such as oral lichen planus, recurrent aphthous ulcer, oral candidiasis and squamous-cell carcinoma. As a microorganism that is beneficial to host health, probiotics can show multi-dimensional therapeutic effects in oral mucosal diseases by targeting and regulating the immune microenvironment of the oral mucosa, inhibiting the colonization of pathogenic bacteria and repairing the barrier function. In this review, we will review the relevant roles that oral-gut microbial homeostasis plays in the pathogenesis of oral mucosal diseases and provide evidence for dysregulation of microbial homeostasis in patients with oral mucosal diseases, and explore specific probiotic strains by regulating oral and intestinal axis microbial homeostasis and host immune and inflammatory responses, in order to provide new ideas for the clinical treatment of oral mucosal diseases.

## 1 Introduction

Oral mucosal disease (OMD) is a variety of infectious and noninfectious diseases that occur in the oral mucosa or oral soft tissue (1). Oral mucosal diseases have a high prevalence worldwide, with the most common recurrent aphthous stomatitis (recurrent aphthous stomatitis) having a prevalence of up to 60% (2). The prevalence of oral mucosal lesions (OML) in children is approximately 5.2% (3) and has now become a true global oral health problem. Oral mucosal diseases are complex in nature, including plaque, papule, blister, erosion, nodule, atrophy and tumor (4), symptomatic treatment is often used.

The oral cavity and gut are the two largest microbial habitats in the human body and play important roles in microbial-related diseases (5). Oral and intestinal microbes are interdependent and jointly participate in the regulation of human physiological functions and pathological processes, which can change the microbial ecosystem of oral and intestinal tracts, thereby inducing the occurrence and development of related diseases (6). In recent years, the oral-intestinal axis has been a hotspot of biomedical research and has been well recognized in a variety of diseases, including gastrointestinal and central nervous system disease (7, 8). More and more evidences show that the microbiota plays a key role in oral mucosal diseases, and the imbalance of some microorganisms directly leads to the occurrence of some oral mucosal diseases. Studies have shown that the oral mucosa has potent immunomodulatory capacity to exert defense mechanisms by protecting or inhibiting the development of oral and systemic diseases (9). This mechanism depends on the role of the oral and gut microbiota in the development and regulation of the local immune system. The microbiota supports the homeostatic construction of the immune system through independent regulatory mechanisms, in which Th17 is involved in immune surveillance and maintenance of mucosal barrier integrity (10, 11). Treg cells also have an indispensable role in maintaining mucosal immune homeostasis (12).

As key supplements for immunomodulatory and balanced microbiota activity, probiotics have been shown to have some efficacy in oral mucosal diseases because they protect the host from pathogens (13, 14). In the oral cavity, probiotics (*Streptococcus salivarius K12* and *Lactobacillus reuteri*) inhibit pathogen colonization, secrete antimicrobial peptides-Nistobacterin, and regulate oral pH mainly through local action, rapidly relieving inflammation (15); Probiotics in the gut (*Bifidobacterium longum* and *Lacticaseibacillus paracasei JY062*) effectively regulate systemic immune balance by regulating intestinal barrier function, generating short-chain fatty acids (SCFAs), and regulating the Th17/Treg cell ratio (12). Therefore, this review aims to clarify the physiological and pathological relationship between oral and intestinal microbes, and summarize the effect and mechanism of probiotics in the treatment of different oral mucosal diseases, it is expected that probiotics can regulate the balance of oral and intestinal microflora in order to treat oral mucosal diseases and provide evidence for clinical treatment of this kind of diseases.

## 2 Oral and gut microbes and oral health

### 2.1 Oral microbiota

The oral microbiota is the second largest microbial community in the human body, comprising a complex microbiota of more than 770 species of bacteria comprising mainly bacteria, protozoa, fungi and viruses (16). The composition of oral microflora showed certain regularity, among which *Streptococcus* dominated, followed by *Fusobacterium, Porphyromonas, Neisseria sicca, Corynebacterium and Actinomycetes*, as well as *Lactobacillus* and others, intraoral microbes have both individual-and site-specific, and buccal and palatal mucosa are areas of low microbial diversity (17, 18).

Commensal oral microbiota can enhance oral mucosal immunity and resist colonization and expansion of pathogenic microbes that cause oral disease through host immunity or direct competition (19). They maintain a balance in oral health through a strategy of occupying the colonized areas as a dominant population and repelling the invasion of pathogens. Studies have shown that certain types of bacteria, including *Streptococcus, Actinomycete* and *Bifidobacterium*, extracted from healthy mouths can effectively inhibit the growth of Porphyromonas. gingivalis, preventing it from overproducing. These symbionts ensure the stability of microbial ecosystems through their competitive advantage in the oral environment, thus favoring overall oral health (20–22). *Lactococcus lactis*, a commensal organism in the oral microbiota, can produce streptococcin lactis that has been shown to reduce the occurrence of oral tumors and prolong the lifespan of tumor mice, in addition, Radaic et al. further showed that the nisin-producing probiotic L. lactis can prevent and destroy oral biofilms, reduce the number of oral pathogens within oral biofilms, and restore the diversity of oral biofilms to controlled levels (15). Recent studies have also shown that nistobacin can attenuate pathogen-induced oral tumorigenesis, cancer cell migration, and cell invasion of oral squamous cell carcinoma *in vitro*, *in vitro*, thus suggesting that commensal microbial species have anticancer effects (20). Although oral microbes exist as symbiotics, maintaining a relationship with the host on the basis of reciprocity and mutual benefit, when they break through the symbiosis barrier, some microbes will transform into pathogens, cause disruption of oral homeostasis or “dysbiosis” (17).

### 2.2 Gut microbiota

The gut microbiota is a complex and diverse ecosystem, characterized by the use of 16S ribosomal RNA amplicons and metagenomic sequencing techniques, thousands of microbes are found to inhabit the gut (23). These microbes co-evolve with the host and have important implications for health and disease. The diversity of gut microbes changes as the body grows, and more and more studies show that the relationship goes both ways, the microbiota can also influence host physiological and pathological processes (24). For example, changes in the gut microbiota can selectively bind to e-cadherin, activating the β-catenin signaling pathway, which may trigger carcinogenesis and inflammatory responses; Increases the risk of colon cancer (25). However, the gut microbiota also has protective and immunomodulatory effects. These microbes function not only through colonization resistance mechanisms, that is, through a range of strategies to reduce the risk of colonization of potential pathogenic bacteria in the gut (26), thus, a healthy and stable state of the gut microbiome is maintained, and some bacteria also possess unique antimicrobial systems capable of effectively controlling bacterial overgrowth and inhibiting the invasion of foreign pathogens (27). In addition, the gut microbiota is involved in the regulation of innate and adaptive immune responses of the host through a variety of complex mechanisms. They help the host immune system to recognize and distinguish itself from outside microbes, timely promoting or suppressing inflammatory responses (24, 28), thereby establishing a dynamic immune balance within the host. This balance is critical for regulating the interaction between TH cells and Treg cells to maintain the stability of the immune system. An in-depth understanding of these functions will facilitate in-depth studies of the role of the regulatory microbiota in health and disease states and provide new strategies for the prevention and treatment of related diseases.

### 2.3 Mouth-gut axis microbiota

The gastrointestinal tract is a continuous tube that extends from the mouth to the anus and is home to many different bacterial communities. These communities interact and occupy different niches throughout the canal. Of these niches, the oral and gut regions are the most abundant (29). The oral-intestinal axis is an emerging area of research (30). The oral-gut barrier is well-positioned to insulate the oral and gut microbiota (31), but several high-throughput sequencing studies (5, 6, 32) have shown that more than half of the microbial species detected at these two sites show evidence of oral-intestinal translocation; The oral microbiota can travel down to the gut, exacerbating various gastrointestinal disorders, and the gut microbiota can also travel up to the oral cavity, affecting the structure of the oral microbiota (33).

Microbial crosstalk in the oral-intestinal axis can affect the occurrence and prognosis of oral mucosal diseases through bidirectional migration and metabolic regulation. Conditionally pathogenic bacteria in the oral cavity (*Fusobacterium nucleatum* and *Porphyromonas gingivalis*) can migrate to the intestine through swallowing or blood circulation, for example, dysbiosis of the oral flora caused by periodontal inflammation can weaken the resistance to intestinal colonization and exacerbate the pathological process of inflammatory bowel disease by disrupting the intestinal barrier function (7). At the same time, gastric *Helicobacter pylori* can ectopically colonize the oral cavity through bloodstream, and its induced inflammatory cytokines (such as IL-8, TNF-α) directly disrupt the oral immune microenvironment and promote local inflammatory responses in diseases such as oral lichen planus (34). For example, SCFAs produced by fermentation of dietary fiber by intestinal flora reach the mucosa through blood circulation and inhibit the over-activated immune response (35), significantly reducing the degree of inflammation in recurrent aphthous ulcers, demonstrating the complexity and therapeutic potential of export-intestinal axis microbes in the regulation of oral mucosal diseases.

## 3 The relationship between oral-intestinal microbes and oral mucosal diseases

### 3.1 Oral mucosal diseases

Oral diseases are an important public health issue, and prevalence, which affects all age groups, has a significant negative impact on quality of life (36). The oral mucosa consists of the lamina propria and submucosa and can be divided into three major groups: masticatory mucosa, inner mucosa and special mucosa (37). The presence of oral mucosal commensal microbiota is critical for promoting and protecting the establishment of homeostatic immune responses, which achieve this function by providing appropriate amounts of pathogenic stimuli. This kind of stimulation can maintain the normal activity of the immune system, while the physical properties of the mucosal layer act as a natural physical barrier to prevent the invasion of external stimuli; The function of mucosal immune barrier makes it possible to recognize microbial antigens and trigger corresponding immune responses (9).

**Figure 1:**
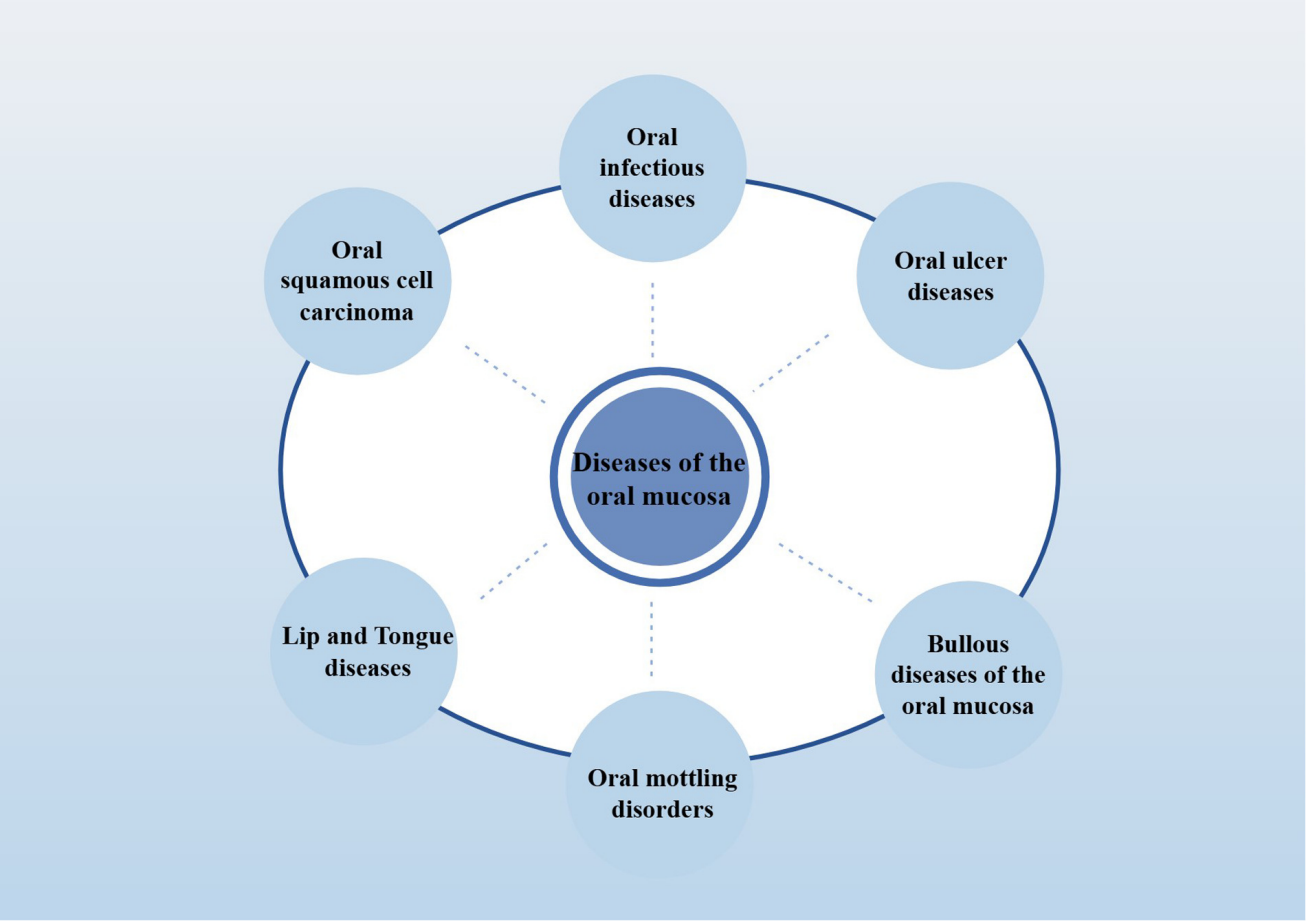
Classification of oral mucosal diseases.

Oral mucosal diseases, common including infectious diseases (including oral candidiasis and oral herpes) and non-infectious inflammatory diseases (including oral lichen planus and recurrent aphthous stomatitis), may pose a significant threat to oral health (38) (The detailed classification is shown in Figure 1; Classification of oral mucosal diseases). In addition, many systemic inflammatory diseases (including: lupus erythematosus, AIDS) also have the typical oral inflammation. At present, the pathogenesis of various oral mucosal diseases is not clear, but most of them are mucosal inflammatory damage, it is characterized by changes in color, local hyperemia, erosion, nodules, pain, or markings on the skin and mucosa, often caused by immune factors, viral, and bacterial infections (39). The ectopic colonization of resident bacteria of the gut, including *E. coli* and *Helicobacter pylori*, is one of the major factors contributing to the exacerbation of oral mucosal lesions (40). Furthermore, saliva and oral microbiota also play a role in the healing process when mucosa is damaged (41). Therefore, the key to the treatment of oral mucosal diseases is to promote the repair of local mucosal wounds and the alleviation of systemic inflammation.

### 3.2 The influence of imbalance of oral and intestinal microbial homeostasis on oral mucosal diseases

Studies have shown that oral ingestion of microbiota may migrate to the digestive tract and trigger a pathogenic immune response (30). The mucous membrane is an important barrier for pathogens to invade the human body. Most antigens or infectious factors penetrate the human body through the mucous membrane, interactions between the mucosal-associated immune system and microbiota are critical for maintaining mucosal tissue homeostasis and preventing inflammatory diseases that occur at mucosal sites (42).

The interaction between the oral microbiota and the immune system and epithelial barrier has been well discussed in the gut (29). However, there are relatively few studies on the ectopic colonization of the oral cavity by intestinal pathogenic bacteria, which can cause oral diseases. Recent studies have revealed that the occurrence of OMD is closely related to the ecological collapse caused by the imbalance of host-microbial interactions, and its microbial composition characteristics vary according to the type of disease, for example, OLP patients present with abnormal proliferation of *Fusobacterium nucleatum* and *Streptococcus*, which aggravates mucosal damage by activating the Th17 pathway and pro-inflammatory factors (43, 44). RAS was significantly associated with *Porphyromonas gingivalis* enrichment and *Lactobacillus reuteri* depletion, which inhibits pathogens and strengthens barrier function by secreting bacteriocin and butyric acid, while lactic acid and hydrogen peroxide produced by *Streptococcus salivarius* reduce the risk of ulceration by regulating the local microenvironment (45, 46). The targeted changes of these specific microbiota and their metabolites not only reveal the pathogenesis of OMD, but also provide a direction for precise intervention, and provide a prerequisite for personalized treatment of OMD.

## 4 The basic principles, mechanisms and potential of probiotics in the treatment of oral mucosal diseases

**Figure 2:**
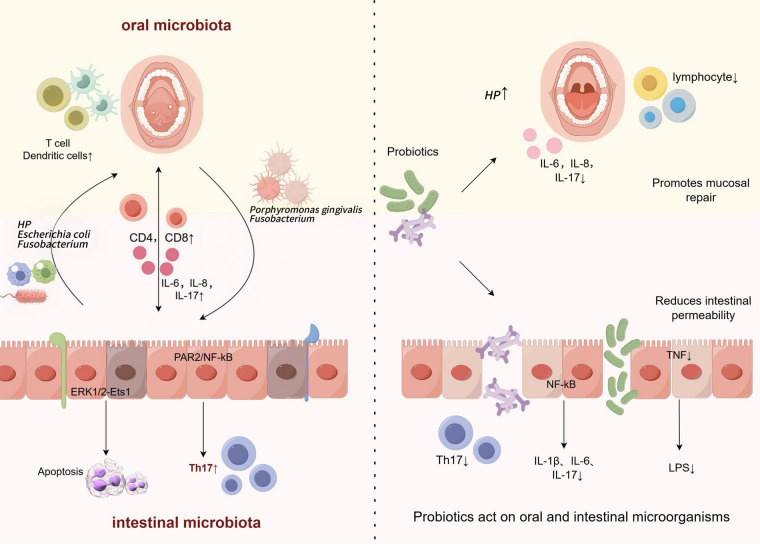
Oral and intestinal microbial relationships and the pathways and potential mechanisms of probiotics in the treatment of oral mucosal diseases.

Probiotics are a group of living microorganisms that provide health benefits to the host when a certain level of intake is achieved. They modulate the host immune response through multiple mechanisms, including microbial competition and inhibition (e.g., competing for colonization sites, secreting antimicrobial substances), reduction of pathogenic microbes, enhancing mucosal barrier repair and function (e.g., promoting tight junction protein expression), reduction of intestinal permeability, and direct effects with the mucosal immune system (Figure 2: Oral and intestinal microbial relationship and the pathway and potential mechanism of probiotics in the treatment of oral mucosal diseases) (47). Furthermore, a key mechanism involves immunomodulation, specifically regulating the balance of pro-/anti-inflammatory factors (e.g., Th17/Treg axis). The immunomodulatory function of probiotics is strain-specific, which means that different strains of probiotics may have different effects on the immune system. In the oral mucosa, probiotics focus on the regulation of the oral immune microenvironment (48), *Porphyromonas gingivalis* activates NF-κB inflammation through TLR4, *Lactobacillus reuteri* inhibits TLR4 phosphorylation and downstream NF-κB/MAPK pathway, reduces IL-6 and IL-8 levels to alleviate RAS, and *Lactobacillus acidophilus* inhibits inflammation by increasing the proportion of Treg cells, Inhibition of Th17 cell-mediated IL-17 secretion corrects the immune imbalance of OLP, and its SCFAs can also inhibit TLR4 signaling to reduce abnormal apoptosis of keratinocytes. In the intestinal mucosa, probiotics (*Bifidobacterium longum and Lactobacillus acidophilus*) can reduce gene expression of NF-κB, IL-17, and TNF pro-inflammatory signaling pathways to significantly reduce inflammatory infiltration and reduce the incidence of precancerous lesions (13, 49). For example *Lactobacillus fermentum XY18* can be reduced by decreasing serum motilin, substance P, and interleukin. Serum somatostatin is raised to alleviate HCl/ethanol-induced gastric injury in mice (50, 51).

The targeted regulation of specific strains shows the therapeutic potential for OMD. These diverse mechanisms—encompassing immunomodulation, microbial competition, and mucosal barrier enhancement—highlight the multi-target characteristics of probiotics in mucosal immune regulation. However, their efficacy is affected by factors such as strain specificity, delivery pathway, and host microenvironment, and further optimization research is needed to achieve precision therapy.

(Left Panel: Pathogenic Crosstalk) Ectopic colonization of the oral cavity by gastrointestinal pathogens (e.g., Helicobacter pylori, Escherichia coli) via retrograde translocation disrupts oral microbial homeostasis. These pathogens synergize with oral residents like Porphyromonas gingivalis to:(i) Drive lymphocyte subset imbalance and elevate inflammatory cytokines (e.g., IL-6, IL-8, IL-17); (ii) Activate immune dysregulation through PAR2/NF-κB and ERK1/2-Ets1 signaling pathways, promoting oral mucosal pathogenesis.) (Right Panel: Probiotic Intervention) Probiotics counter disease progression by: (i) Suppressing pro-inflammatory signaling (NF-κB, TNF, IL-17) to reduce inflammatory infiltration;(ii) Preserving epithelial barrier integrity via prevention of tight junction protein degradation (e.g., by reducing LPS burden); (iii) Modulating ectopic pathogen colonization and restoring oral microbial equilibrium, thereby attenuating inflammatory biomarkers and improving the mucosal microenvironment.)

## 5 Probiotics for the treatment of oral mucosal disease

Oral mucosal diseases are a common oral health problem whose pathogenesis is related to oral microbiota imbalance, immune disorders and chronic inflammation. In recent years, probiotics have gradually become a research hotspot in the treatment of oral mucosal diseases due to their role in regulating microbial homeostasis and immune homeostasis. Several randomized controlled trials (RCTs) have used probiotics as an intervention in the experimental group to explore their efficacy in diseases such as oral lichen planus and recurrent oral ulcers (Table 1). These studies have preliminarily confirmed that probiotics can improve oral mucosal diseases by inhibiting pathogenic bacterial colonization, regulating immune responses, and reducing inflammation levels.

**Table 1:** Randomized studies of the use of probiotics as an experimental group in oral mucosal diseases.

Source	Study design	Sample size (n)	Probiotic strain	Form and dosage of probiotics	Study duration	Results	Conclusion	Reference
Marlina et al. (52)	Proof of concept, parallel group, randomized placebo control	40 OLP patients (15 placebo, 15 probiotics)	*VSL#3* (Multi-strain probiotic brand)	Twice a day, a bag or two particles	30 days	Both VSL#3 and placebo groups had similar reductions in mean pNRS scores at 30 days, with no evidence of a statistically significant difference between the random groups (*p* = 0.749).	High concentrations of probiotic VSL# 3 are safe and has good tolerance in patients with OLP	(52)
Xia et al. (53)	Randomized, double-blind, placebo-controlled	77 nasopharyngeal carcinoma patients with oral cavity mucositis induced by radiation and chemotherapy with (1:1) probiotic mixture: placebo	Oral probiotic mix (*Lactobacillus plantarum MH-301109CFU, B.nimalis LPL-RH109CFU, Lactobacillus rhamnosus LGG-18109CFU and Lactobacillus acidophilus 109CFU*)	2 times a day, one at a time capsule	7 weeks	Morbidity was reduced in the probiotics group compared with the placebo group, and patients in the probiotic cocktail group had reduced rates of depletion of CD3T cells, CD4T cells, and CD8T cells	Improvement of a probiotic mixture by enhancing an immune response in patients with nasopharyngeal carcinoma (NPC) and changing the structure of intestinal flora, significantly reduces the severity of the OM	(53)
Villar-García et al. (54)	Randomized, double-blind, placebo-controlled	Forty-four patients with noncontiguous HIV-1 infection with viral load <20copies per milliliter for at least 2 years (oral probiotic supplement: placebo = 1:1)	*Brady's streptococcus*	Two capsules were given 3 times a day capsule	12 weeks	Differences were observed between the probiotic and placebo groups for LBP values (−0.30 vs. +0.70 pg/ml) and IL-6 (−0.60 vs. +0.78 pg/ml)	Add probiotics (Brady's streptococcus) treatment can reduce long-term virologic suppression of infection in patients with HIV-1 microbial translocation (LBP) and inflammatory parameters (IL-6)	(54)
Bjarnason et al. (55)	Randomized, double-blind, placebo-controlled	62 patients with CD (29) 33 people, probiotics placebo	Many strains of probiotics Symprove (*Rhamnose lactobacillus NCIMB30174 NCIMB30173, lactobacillus acidophilus, lactobacillus, plant NCIMB30175 and excrement enterococcus NCIMB30176*)	1 ml/kg/day) liquid	4 weeks	FCAL levels were significantly lower in UC patients receiving probiotics compared with placebo (*p* < 0.015).	There were no significant differences between patients treated with probiotics and those treated with placebo, and no patients experienced clinical relapse	(55)
Li et al. (56)	Randomized controlled trial	65 patients with candida-associated oral mucositis (probiotics +2% sodium bicarbonate solution and 2% nystatin cream: 2% sodium bicarbonate solution and 2% nystatin cream = 1:1)	Local probiotics (*Long bifidobacterium, a mixture of streptococcus thermophilus and lactobacillus Bulgaria*)	Four tablets three times a day lozenge	4 weeks	The detection rate of Candida was lower in both groups (*P* = 0.000), and the probiotics group was significantly lower than the control group (*P* = 0.038).	Topically administered probiotics help improve some clinical conditions and reduce the prevalence of Candida SPP	(56)
Samiraninezhad et al. (57)	Randomized, double-blind, controlled	60 patients with oral ulcer (probiotics group: = 1:1 control group)	Chitosan nano gel/probiotic mixture (*Lactobacillus reiki* probiotic suspension)	Injections three times a day Injection agent	1 week	The lesion size and pain intensity probiotic group were reduced	The use of a topical probiotic nanoformulation containing Lactobacillus reiki reduced lesion size and pain severity more rapidly than topical analgesic mouthwashes in patients with RAS	(57)
Vesty et al. (58)	Randomized, double-blind, placebo-controlled trial	13 patients with head and neck cancer after radiotherapy (7 on probiotics and 6 on placebo)	Oral probiotic lozenges (Containing *Streptococcus saliva M18*)	3 times a day	4 weeks	Probiotics group and placebo group no plaque was observed between teeth surface significantly reduced (*p* > 0.05)	Oral probiotic lozenges to no significant impact on bacterial community composition and diversity	(58)

### 5.1 Oral infectious diseases

#### 5.1.1 Hand, foot and mouth disease

Hand, foot and mouth disease (HFMD) is a viral disease that predisposes to children in Asia and is associated with infectious, seasonal disease (59, 60), the clinical manifestations are herpes and rash on the mouth and limbs, and the pathogens are mainly enterovirus A71(EV71) and Coxsackie virus A16(CVA16) (61). Currently, there are no specific pharmacological interventions for HFMD, and vaccines remain highly protective against EV-A71-related HFMD (59). However, because of the concurrent spread of multiple pathogens and the dynamic changes in molecular epidemiology of infectious pathogens, interventions that rely solely on a single pathogen are relatively inadequate, the key to control this disease is to find safe and effective drug treatment and prevention. Probiotics are widely used to complement nutritional strategies aimed at enhancing intestinal immunity, and the efficacy of treating hand, foot and mouth disease in children is unquestioned (62), it can maintain intestinal immunity and anti-inflammatory reaction by decreasing the concentration of serum pro-inflammatory factors il-1β, IL-6, TNF-α, INF-*γ*, increasing the diversity of *α* and the abundance of bacteria in gastrointestinal tract. In addition, it was found that the content of *Roseburia inulinivorans* and *Romboutsia timonensis* was high after treatment, it is therefore inferred that both are promising potential probiotics for the treatment of HFMD (62, 63). However, in a study by Guo X and colleagues, although probiotics combined with prebiotics have been shown to be effective in the treatment of hand-foot-mouth disease, the exact type and dose of the combination remain unclear, and there is potential for bacterial resistance in children who take this combination. Therefore, the presence of this resistance in the gut microbiota should be validated in future follow-up studies, repeated and larger populations were sampled before and after infection to confirm that probiotics with prebiotic binders are able to reduce susceptibility to HFMD by modulating the gut microbiota (64).

#### 5.1.2 Oral candidiasis

Oral candidiasis (OC) is one of the most common fungal infections of the Oral cavity, it occurs in different forms of pain: pseudomembrane, erythema, or increment (36), often manifested as dry mouth, sticky, oral mucosa burning sensation, taste, pain, in the oral ecological imbalance environment, Candida species of yeast overgrowth and become toxic, changes in oral microbiota diversity and composition driven by systemic and local susceptibility factors are secondary (65, 66). A recent systematic analysis confirmed that the specific pathway of probiotic therapy is to inhibit the growth of Candida albicans, an experimental conclusion that suggests that probiotic therapy is promising to change current clinical guidelines for the prevention or treatment of oral candidiasis, especially for immunocompromised patients and patients with refractory, drug-resistant or recurrent infections, *lactobacillus rhamnosus GG (LrGG)* and *Lactobacillus reuteri* are expected to be the targeted probiotics in oral environment, which can reduce the number of oral candida species. LrGG bacteria have the ability to adhere to epithelial cells, promote type 1 immune response, and through inhibiting the activation of macrophages to effectively reduce the inflammatory response, and promote the production of innate immune cells such as IL-10, IL-12, and TNF-α (67, 68). *Lactobacillus reuteri*, on the other hand, cooperates in changing the PH of the oral environment through the colony effect and the production of lactic acid and other acidic substances, simultaneous generation of H2O2 to effectively reduce overgrowth and potential toxic effects of Candida *in vivo* (65, 69). Future research should focus on the differences of oral microflora and the relationship between oral microflora and the host in order to achieve personalized probiotics control strategies. These advances will help promote probiotics as a safe alternative to conventional fungicide in oral candidiasis.

### 5.2 Oral ulcer-like diseases

#### 5.2.1 Recurrent aphthous ulcer

Recurrent aphthous ulcer (RAU), also known as recurrent aphthous stomatitis or recurrent oral ulcer, is the most common oral mucosal ulcer disease that severely affects patients' oral health and quality of life (70). The etiology and pathogenesis of RAU are unclear, and the diagnosis is based primarily on the characteristics of the history (recurrent, self-limited) and clinical features (concave, and pain) (36, 71). At present, there is no specific method to cure RAU. The treatment is mainly symptomatic, aiming at relieving pain, promoting ulcer healing and prolonging the interval of recurrence. At present, the treatment of RAU mainly focuses on the long-term use of steroids to relieve symptoms, promote ulcer healing and prevent recurrence, however, it has certain limitations and can lead to atrophy of the oral mucosa and immunodeficiency (72). Pedersen, A studied the effect of probiotic supplementation on the severity of aphthous ulcer lesions over 3 months in patients with RAU and found that daily supplementation with *Lactobacillus reuteri* reduced the severity of aphthous lesions over 90 days, however, there was no advantage compared with placebo and the effective practice of probiotic treatment of RAU was brief (73, 74). It should be noted, however, that some recent studies have reported conflicting results, with Nirmala finding reduced ulceration and pain in patients after topical application of *Bacillus cloacae*, therefore, probiotics have been suggested as an adjunct to the treatment of recurrent aphthous stomatitis; Samiraninezhad, N. indicated that treatment of RAU with nano-preparations of *Lactobacillus reuteri-derived probiotics* has sustained efficacy and may be a promising therapeutic option for RAU (57). The clinical efficacy of *Lactobacillus reuteri* in the treatment of RAU is significantly different due to the treatment observation time (the former may be due to long-term follow-up due to insufficient strain colonization ability or host adaptability changes leading to weakened effect) and drug delivery mode (the latter combined with nano-drug delivery technology may improve the residence time and targeting of probiotics in the oral mucosa), suggesting that future studies need to standardize the observation period and combine advanced delivery technologies to more accurately assess the long-term therapeutic potential of specific probiotics.

#### 5.2.2 Traumatic blood blisters/traumatic ulcers

The occurrence of oral traumatic ulcer is mostly due to the chronic mechanical stimulation of oral epithelial tissue. When the stimulating factors are strong and the body responds quickly, traumatic blood blister will occur, when the irritant persists and the patient is not well treated, it further evolves into oral squamous-cell carcinoma (75). The treatment of a traumatic ulcer or blister is usually the use of preservatives, antibiotics, and steroids (76). However, prolonged use of antibiotics and steroids can cause drug resistance and unpleasant side effects (70). The disease is treated primarily to promote wound healing, which is associated with fibroblasts and surrounding blood vessels. Several trials have shown that probiotics can help heal traumatic ulcers, the mechanism may be that probiotics promote the migration and proliferation of fibroblasts and induce the production of IL-8, which stimulates the migration of endothelial cells to form new blood cells, it is not discussed that probiotics as “Active microorganisms” have a good immunomodulatory effect on oral and gastrointestinal flora. A study by Kusumaningsih et al., in addition to obtaining the same results as above, also found that probiotics interact with the gastrointestinal mucosa during inflammation during ulcer development, activation of the TLR pathway inhibits proinflammatory cytokine production, thereby increasing the gastrointestinal mucosal barrier, modulating the immune system, and accelerating the repair of traumatic ulcers (76); The results of this study clarify the specific mechanism and potential therapeutic effect of probiotics in the treatment of traumatic ulcer, and provide a new idea and method for the treatment of traumatic ulcer.

#### 5.2.3 Radiation stomatitis

Radiation therapy-induced oral mucositis (RIOOM) is a disease of the oral mucosa characterized by oral ulcers in cancer patients treated with head and neck radiotherapy and chemotherapy (CRT) (77), oral mucositis is also a common side effect in cancer patients treated with chemoradiotherapy or hematopoietic stem cell transplantation (78). At present, a variety of drugs are used to treat the disease, including ibuprofen, biandamine mouthwash, oral honey or Chinese herbal preparations, which appear to be safe, pain associated with mucositis can be relieved (79–81, 107). A recent study showed that dysbiosis of the oral microbiota underlies the development of mucositis, so whether the application of probiotics to modulate the microbiota to treat RIOOM is effective has received widespread attention. Jiang and Xia et al. studied the effect of probiotics on oral mucositis in patients with nasopharyngeal carcinoma (NPC) after radiotherapy and chemotherapy, from the incidence and degree of OM and the results of high-throughput sequencing, it is inferred that the combination of probiotics may improve OM by improving patients' immunity. After two years of observation and experiments, they found that chemoradiotherapy affected epithelial cells by inducing DNA and non-DNA damage, activated reactive oxygen species and activated TLR4/NF-ΚB pathway, promoted the production of TNF-α, IL-1β and IL-6, and accelerated apoptosis, in addition, TNF-A, IL-6, IL-1β, and apoptosis play a central role in the development of mucositis, and IL-6 levels are positively correlated with the severity of OM in radiotherapy patients taken together (53), probiotic mixtures can reduce TLR4/NFΚB activity and slow inflammation and apoptosis induced by chemotherapy and radiotherapy.

### 5.3 Oral mucosal bullous disease

#### 5.3.1 Pemphigus vulgaris

Pemphigus is a rare autoimmune vesicular disease in which IgG autoantibodies inhibit the adhesion function of desmosomal core proteins (108), resulting in loss of cell adhesion capacity of keratinocyte; Induces vesicular formation (82). Pemphigus vulgaris (PV) is the most common and serious autoimmune bullous disease in Pemphigus vulgaris, which is characterized by the formation of flaccid blisters and erosive lesions on the skin or mucosa The occurrence of PV has been shown to be associated with dysbiosis of both the oral and gut microbiota (83, 84). Wang et al. further systematically explored the gut microbiota and metabolites of PV patients by metagenomic shotgun sequencing and targeted metabolomics approaches, the diversity of the gut microbiota in PV patients was found to differ from healthy family members, with some pathogens, such as *E. coli*, having higher relative abundance in PV patients; These pathogens may cause PV by disrupting the balance of the gut microbiota (85). Although this study has demonstrated an association between the gut microbiota and PV onset, to date, there is no evidence that the use of probiotics has a role in the treatment of this disease. Therefore, further targeted studies are needed to assess whether supplementation with probiotics or probiotic derivatives is a useful strategy for the treatment of PV.

#### 5.3.2 Oral mucosal pemphigoid

Oral mucosal pemphigoid (OMMP) is one of the most common forms of pemphigoid, which is an autoimmune subepithelial vesicular disease (86), 80% of the cases were oral mucosal Diseases, which often manifested as recurrent blistering of the gums for several months or years, which could not be cured, there is no significant racial difference.

MMP is relatively difficult to diagnose clinically and its pathogenesis is poorly understood, but there is evidence that it is a complex autoimmune disease mediated by autoantibodies targeting different components of the basement membrane, cellular immunity is also involved in the pathogenesis and treatment is usually aimed at relieving pain, which may include corticosteroid and immunosuppressive agents. However, the use of these drugs may be ineffective (87). A study of oral mucosal pemphigoid combination therapy has given life to the treatment of OMMP, Santonocito et al. compared clobetasol propionate oral gel 0.05% local treatment, niacinamide, oral probiotics and doxycycline four ways of treatment, found that the four ways to improve the symptoms of MMP patients were improved, corticosteroid, doxycycline, and nicotinamide are all good at controlling local reactions and inflammation, and probiotics can balance the dysbacteriosis of the oral cavity without adverse reactions, based on this evidence, let us focus on probiotics as an important adjunct to first-line therapy in the adjuvant and long-term treatment of MMP (82).

### 5.4 Oral stripe diseases

Oral lichen planus (OLP) is a common chronic inflammatory disease of the oral mucosa with a global prevalence of 1.01% (88). The pathogenesis of OLP is thought to involve dysregulation of the immune system, including presentation of unknown antigens, activation and migration of T lymphocytes, production of proinflammatory cytokine and chemokines leading to subepithelial inflammatory infiltration; Keratinocyte damage and disruption of epithelial homeostasis (89–91). Clinically, the disease presents as hyperkeratosis reticularis of the oral mucosa, with long-term erosions and ulcers occurring in most individuals, leading to pain and dysfunction leading to decreased quality of life (92). At present, there is no cure method, the first-line drug corticosteroid has produced side effects and clinical effect is not stable, so it is important to find a long-term and safe therapeutic supplement.

Probiotics are a well-established biological immunomodulator that can induce the ability of Tregs to suppress autoimmunity through different mechanisms (93) currently, clinical trials and systematic reviews conducted by probiotics for the treatment of OLP are abundant, but there is little strong evidence to support the use of probiotics for the treatment of OLP (94, 95). A proof-of-concept, parallel-group, randomized, placebo-controlled study (clinical and biological effects of probiotics in patients with oral lichen planus, Cabrio) conducted by Marlina, E, and colleagues led us in a new direction, they used Cabrio to assess the feasibility and safety of high concentrations of a probiotic mixture (*VSL # 3*) in the treatment of oral lichen planus, and concluded that high concentrations of the probiotic *VSL # 3* were safe in the treatment of OLP, and well tolerated (52). This suggests that our use of probiotics in the treatment of OLP may be effective, but targeted probiotics need to be sought rather than the abuse of probiotics.

### 5.5 Lip and tongue disease

Burning Mouth Syndrome (BMS) is a group of syndrome which mainly occurs on the tongue and mainly presents as burning pain, no characteristic histopathological changes (96). Currently, there is still no universally accepted definition and no appropriate diagnostic criteria, and the prevalence of burning mouth syndrome is estimated to be 2.5%–5.1% in the general population (97). To date, there have been no specific studies applying probiotics to the treatment of burning mouth syndrome. However, there is considerable evidence that dysbacteriosis is an important factor in the occurrence and development of burning mouth syndrome (109). Lončar-Brzak B and colleagues, based on the commonality between periodontitis and burning mouth syndrome, probiotics have a stimulating effect on the taste of the oral mucosa, as well as unpublished articles that found probiotics useful for the treatment of burning mouth, a long-term randomized controlled trial was conducted in which 62 of the 80 included patients completed treatment and underwent follow-up control examinations, oral probiotics and low-intensity laser therapy (LLLT) were demonstrated to be the most effective treatments to improve quality of life (98). Thus, the application of probiotics appears to have a potential role in the prevention and treatment of BMS. However, more clinical studies are needed to elucidate their efficacy and stability.

### 5.6 Squamous-cell carcinoma of the oral cavity

Previous studies have linked dysbacteriosis to different gastrointestinal cancers, carcinogens can be consumed or produced by metabolic activity of microbes present in the gastrointestinal system (99, 100). The role of microbial dysbiosis in oral squamous-cell carcinoma (OSCC) is also increasingly recognized, but few studies have evaluated the microbial composition of OSCC subjects; To explore whether the microbiota of patients and healthy individuals differs, Mäkinen and colleagues used *A.syzygii* to investigate its prophylactic effect on oral squamous-cell carcinoma cell lines and its effect on the salivary microbial profile, the results showed that the appearance of OSCC was associated with an increase in the abundance of angina. In addition, *A. syzygii* secretion has similar anticancer activity compared to cisplatin, and this anticancer substance may be an exopolysaccharide (101). Several subsequent studies have concluded that *Porphyromonas gingivalis* and *Fusobacterium nucleatum* may be promoters of OSCC tumorigenesis, because *Porphyromonas gingivalis* reduces extracellular arginine deiminase protein production by Streptococcus and S. intermedius, low abundance oral bacteria may interact through multiple intermediate pathways, for example, heteromammary fermentation affects the growth of certain bacteria and results in dysbiosis of the oral microbiota, which in turn promotes the development of OSCC (102, 103).

**Figure 3:**
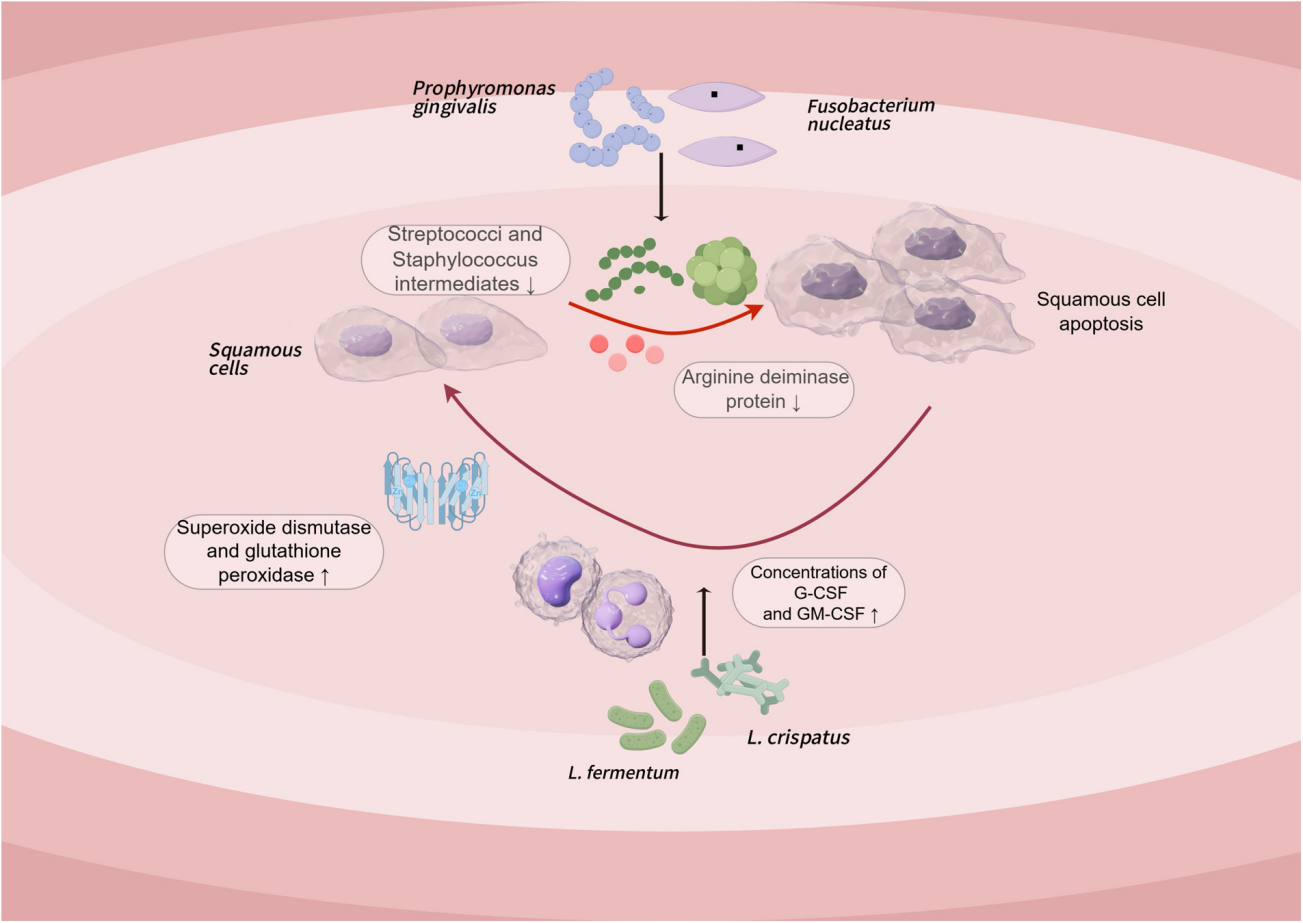
Pathogenesis of oral squamous cell carcinoma and probiotic treatment pathways.

All in all, Probiotics can reduce exposure to carcinogens by detoxifying, producing metabolites that cause apoptosis, stimulating immune responses, and altering gut environmental conditions to inhibit tumor cells (48). An *in vivo* study in mice demonstrated that Lactobacillus has a function of increasing apoptosis and decreasing proliferation of colorectal cancer cells (104). This raises concerns about its potential application in the treatment of oral cancer. In the study of squamous-cell carcinoma of the oral cavity, two well-known lactobacillus species, *L. fermentum* and *L. Crispatus*, it has been shown to increase the concentrations of anti-cancer cytokines G-CSF and GM-CSF, thus preventing the immune decline triggered by tongue cancer, and this Lactobacillus also enhances the activities of superoxide dismutase and glutathione peroxidase, and reduced malondialdehyde concentrations in tissue samples from animal models of tongue cancer, thus beating oxidative stress damage in the tissue (Figure 3: Pathogenesis of oral squamous cell carcinoma and probiotic treatment pathways) (105). In addition, some studies have shown that probiotics may attenuate chemotherapy-associated oral mucositis and improve intestinal barrier function in patients with OSCC (106). It is important to note that side effects such as immune system overreaction, bacteremia, or local infection have not been reported in these studies.

The research on the treatment of OMD with probiotics has gradually moved from the exploration of basic mechanisms to clinical applications, and its core advantage lies in the regulation of mucosal immune balance and microbial homeostasis through multiple targets. Currently, specific strains such as *Lactobacillus reuteri* alleviate RAU by modulating immune mechanisms (73, 74); At the same time, Lactobacillus reuteri can synergistically change the pH of the oral environment and effectively reduce the number of Candida albicans through the cluster effect and the production of lactic acid and other substances (65, 69); Probiotic mixture (*VSL#3*) alleviates inflammatory damage to OLP (52). However, there is significant heterogeneity in the results of existing studies, taking *Lactobacillus reuteri* as an example, the difference in efficacy may be due to the functional heterogeneity of strains, the limitations of delivery regimens (nanodosage forms are more conducive to local oral colonization than oral capsules), and inconsistent efficacy evaluation criteria (some studies use ulcer healing time as the endpoint and ignore long-term indicators such as recurrence rate). In the future, it is necessary to build a standardized strain library (based on genomic and functional omics screening), develop an oral directional delivery system, and integrate multi-omics testing (microbiome, metabolomic, and immunophenotypic analysis) to achieve precise stratified treatment, and explore predictive biomarkers to optimize patient matching strategies to promote the transformation of probiotics from adjuvant therapy to individualized targeted intervention.

## 6 Conclusions and outlook

The imbalance between the microbiota and the host is the basis for the pathogenesis of oral mucosal diseases. Although there are more than 10 types of oral mucosal diseases according to the characteristics of lesions, their pathogenesis is mostly related to microbial homeostasis. As an important microorganism that regulates the microbiota, probiotics can promote oral health by regulating various functions such as oral-intestinal microbiota and host immunity.

This paper summarized the microbial changes in different types of oral mucosal diseases, and discussed the efficacy and mechanism of probiotics in the treatment of oral mucosal diseases. Current studies have shown that the imbalance between the microbiota and the host is an important factor in the pathogenesis of oral mucosal diseases, and its pathogenesis is mostly related to the imbalance of microbial homeostasis. In the treatment of oral infectious diseases, radiation stomatitis, pemphigoid of oral mucosa, burning mouth syndrome and oral squamous-cell carcinoma, probiotics have shown clear efficacy, the main mechanisms are as follows: a. Maintain immune cell immunity and anti-inflammatory response by decreasing serum pro-inflammatory factor, tumor necrosis factor and interferon to increase *α* diversity; b. Promote fibroblast and peripheral angiogenesis to repair local mucosal wounds, c. Competitive colonization of mucosa to prevent the formation of pathogenic bacteria and maintain healthy microbial remission.

However, the study of probiotics in the treatment of oral mucosal diseases still faces three major limitations: a. insufficient clinical evidence, which is manifested by small sample size, low statistical power, and lack of long-term follow-up data; b. Efficacy is inconsistent, the therapeutic effect of different strains on the same disease is significantly different, which may be related to strain specificity; c. Safety issues, the use of probiotics in immunocompromised patients (such as after chemotherapy) may increase the risk of bacteremia, and strains need to be strictly screened (such as excluding strains carrying drug resistance genes).

Recent differentiation of iPSC-derived oral mucosa organoids and the structural similarities between oral mucosa and intestinal epithelium provide new tools for studying simulated disease microenvironments. Future research could integrate metagenomic sequencing and organoid microfluidic models to reveal strain-specific mechanisms; clinically, rigorous reporting and comparison of specific strains, optimization of dosing regimens, and exploration of optimal delivery routes should be prioritized to advance translation. Clinical designs need to increase sample size, adopt stratified randomized controlled trials, and combine dietary interventions with local delivery systems. Furthermore, it is essential to establish a specific efficacy assessment system for OMD that considers dynamic changes in the microbiome and immune markers to accelerate the clinical translation of personalized probiotic therapies from bench to bedside.
